# Characterization of chromosome constitution in three wheat - *Thinopyrum intermedium* amphiploids revealed frequent rearrangement of alien and wheat chromosomes

**DOI:** 10.1186/s12870-021-02896-9

**Published:** 2021-03-04

**Authors:** Yu Cui, Piyi Xing, Xiaolei Qi, Yinguang Bao, Honggang Wang, Richard R.-C. Wang, Xingfeng Li

**Affiliations:** 1 State Key Laboratory of Crop Biology, College of Agronomy, Shandong Agriculture University, Tai’an, 271018 Shandong China; 2 Tai’an Academy of Agricultural Science, Tai’an, 271000 China; 3 USDA-ARS Forage & Range Research Laboratory, Logan, UT 84322-6300 USA

**Keywords:** *Th. Intermedium*, Amphiploid, In situ hybridization, Molecular markers

## Abstract

**Background:**

*Thinopyrum intermedium* (2n = 6x = 42) is an important wild perennial *Triticeae* species exhibiting many potentially favorable traits for wheat improvement. Wheat-*Th*. *intermedium* partial amphiploids serve as a bridge to transfer desirable genes from *Th*. *intermedium* into common wheat.

**Results:**

Three octoploid *Trititrigia* accessions (TE261–1, TE266–1, and TE346–1) with good resistances to stripe rust, powdery mildew and aphids were selected from hybrid progenies between *Th*. *intermedium* and the common wheat variety ‘Yannong 15’ (YN15). Genomic in situ hybridization (GISH), fluorescence in situ hybridization (FISH) and multicolor GISH (McGISH) analyses demonstrated that the three octoploid *Trititrigia* possess 42 wheat chromosomes and 14 *Th. intermedium* chromosomes. The 14 alien (*Th. intermedium*) chromosomes belong to a mixed genome consisting of J-, J^S^- and St-genome chromosomes rather than a single J, J^S^ or St genome. Different types of chromosomal structural variation were also detected in the 1A, 6A, 6B, 2D and 7D chromosomes via FISH, McGISH and molecular marker analysis. The identity of the alien chromosomes and the variationes in the wheat chromosomes in the three *Trititrigia* octoploids were also different.

**Conclusions:**

The wheat-*Th. intermedium* partial amphiploids possess 14 alien chromosomes which belong to a mixed genome consisting of J-, J^S^- and St- chromosomes, and 42 wheat chromosomes with different structural variations. These accessions could be used as genetic resources in wheat breeding for the transfer of disease and pest resistance genes from *Th. intermedium* to common wheat.

**Supplementary Information:**

The online version contains supplementary material available at 10.1186/s12870-021-02896-9.

## Background

*Thinopyrum intermedium* (Host) Barkworth & D.R. Dewey [syn. *Agropyron intermedium* (Host) Beauvor and *Elytrigia intermedia* (Host) Nevski] (2n = 6x = 42; genome formula **E**^**e**^**E**^**e**^**E**^**b**^**E**^**b**^**StSt, JJJ**^**S**^**J**^**S**^**StSt, EEVVStSt** or **J**^**r**^**J**^**r**^**J**^**vs**^**J**^**vs**^**StSt**) is considered as a segmental autoallohexaploid, and its genome constitution hanging in doubt is a research hot subject in *Triticeae* crop research. The presence of **S** genome (later changed to **St** by Wang et al. [1]) in *Th. intermedium* was first reported by Liu and Wang [2] and confirmed by Zhang et al. [3], leading to the genome formula **JJJJStSt** or **EEEEStSt**, where **J** genome is from *Th. bessarabicum* (2n = 14, **JJ** or **E**^**b**^**E**^**b**^), **St** genome is from *Pseudoroegneria strigosa* (2n = 14, **StSt**) and **E** genome is from *Th*. *elongatum* (2n = 14, **EE** or **E**^**e**^**E**^**e**^). Also by using genomic in situ hybridization, Chen et al. considered that the genome constitution of *Th*. *intermedium* is **JJJ**^**S**^**J**^**S**^**StSt** where **J**^**S**^ might be a modified **J** or **E** [4]. Ji et al. concluded that the genome constitution of *Th*. *intermedium* is **E**^**e**^**E**^**e**^**E**^**b**^**E**^**b**^**StSt** by using multicolor GISH (McGISH) [5]. Analyzing the chloroplast *trn*L-F sequence, granule-bound starch synthase I (GBSSI) and GISH, Mahelka et al. (2011) inferred that genomes of *Th*. *intermedium* are related to *Ps*. *strigosa* (**StSt**), *Dasypyrum villosum* (2n = 14, **VV**), and a complex origin from *Th*. *elongatum* (**EE**) and *Aegilops tauschii* (2n = 14, **DD**) [6]. **J**^**r**^**J**^**r**^**J**^**vs**^**J**^**vs**^**StSt** was proposed by Wang et al., who made use of EST-SSR markers to analyze the genome evolution of *Th*. *intermedium*, and **J**^**r**^ and **J**^**vs**^ refer to ancestral genomes of **J**^**e**^(**E**) **and J**^**b**^(**J**), respectively [7].

*Th*. *intermedium* is described as one of the most important perennial Triticeae species, possessing many potentially favorable traits for wheat improvement [8–11]. Due to its crossability with common wheat, the transfer and utilization of *Th. intermedium* disease resistance genes, including the leaf rust resistance gene Lr38 [12]; the stem rust resistance gene Sr44 [13]; the stripe rust resistance gene Yr50 [14]; and the barley yellow dwarf resistance genes Bdv2 [15], Bdv3 [16] and Bdv4 [17, 18], have been accomplished.

*Trititrigia* octoploids were developed from hybrid progenies between *Th*. *intermedium* and common wheat and inherited numerous excellent traits from *Th*. *intermedium*, such as resistance to powdery mildew, leaf rust, stem rust and stripe rust [19, 20]. Furthermore, it is easier to successfully cross common wheat with *Trititrigia* octoploids than with *Th*. *intermedium*. Thus, *Trititrigia* octoploids are valuable germplasm to hybridize with common wheat, continually producing wheat-alien addition, substitution and translocation lines that can transfer beneficial traits from alien species into wheat [9, 21, 22].

Many *Trititrigia* octoploids have been produced in the past five decades and used as intermediary parents to facilitate the transfer of excellent genes from *Th*. *intermedium* to wheat. These *Trititrigia* octoploids include TAF46 [23], 78,829 [24], the ‘Zhong’ series of *Trititrigia* octoploids [25] and the TE series of *Trititrigia* octoploids [20, 21, 26–28]. The TE series of *Trititrigia* octoploids was developed from hybrid progenies between *Th*. *intermedium* and the common wheat variety ‘Yannong 15’ (YN15) and inherited many characteristics from *Th*. *intermedium*, such as large spikes, multi-florets, multi-tillers, and good resistance to stripe rust, powdery mildew and leaf rust. Thus, this TE series of *Trititrigia* octoploids represent an excellent genetic resource for wheat improvement [21, 26–29].

Genomic in situ hybridization (GISH) is generally used to distinguish alien chromosomes in the common wheat background [30, 31]. When *Pseudoroegneria strigosa* (2n = 2x = 14, **StSt**) genomic DNA was used as a probe in GISH analysis of *Th. intermedium*, the karyotype of *Th. intermedium* chromosomes could be classified into three groups. The first group is the **St** genome, completely labeled by probe signals, the second group is the **J**^**S**^ genome labeled only in the centromere areas, and the third group is the **J** genome labeled only in the subtelomeric position [4, 32]. As GISH with the **St**-genome DNA probe could effectively distinguish the three sub-genome of *Th. intermedium*, it was widely used in the identification of *Th. intermedium*-derived wheat germplasms [19, 21, 28, 33].

Fluorescence in situ hybridization (FISH) and multicolor GISH (McGISH) are generally used to distinguish different chromosomes, analyze the chromosomal constitution and detect chromosomal variations in cytological studies [19, 20, 34, 35]. The available FISH probes that reveal fine banding patterns in wheat chromosomes include the *Aegilops tauschii* clone pAs1 [36, 37], the rye clone pSc119.2 [38], the GAA-satellite sequence [39], *T. aestivum* clone pTa535 [40] and many other oligonucleotides [41, 42]. Wheat chromosomes can be clearly distinguished using a combination of FISH probes including the pAs1 clone and the GAA-satellite sequence. Chromosome painting using this combination of FISH probes has been achieved in the common wheat cultivar ‘Chinese Spring’ (CS) [43, 44] in which all chromosomes of A, B and D subgenomes were labeled with specific signals, with the signals on the B- and D-genome chromosomes being particularly abundant. Through combined McGISH and FISH analysis, the positions and characteristics of chromosomal structural variations in such materials can be revealed.

In this study, the chromosomal constitution and chromosomal variations in three *Trititrigia* octoploids (TE261–1, TE266–1 and TE346–1) exhibiting good resistance to stripe rust, powdery mildew and aphids were investigated. The genetic characteristics, types of chromosome variation of the three *Trititrigia* octoploids were revealed using GISH, FISH, McGISH and molecular markers.

## Results

### Chromosomal constitution of the three *Trititrigia* octoploids

The root tip chromosome analysis showed that chromosome number of TE261–1, TE266–1 and TE346–1 was 2*n* = 56. Observation of meiotic chromosomes in PMCs revealed that most chromosomes in the observed cells formed 28 bivalents at meiotic metaphase I, 14 alien chromosomes formed 7 bivalents were observed in GISH analysis, indicating high cytological stability (Supplemental Fig. 1, Supplemental Table 1).

**Fig. 1:**
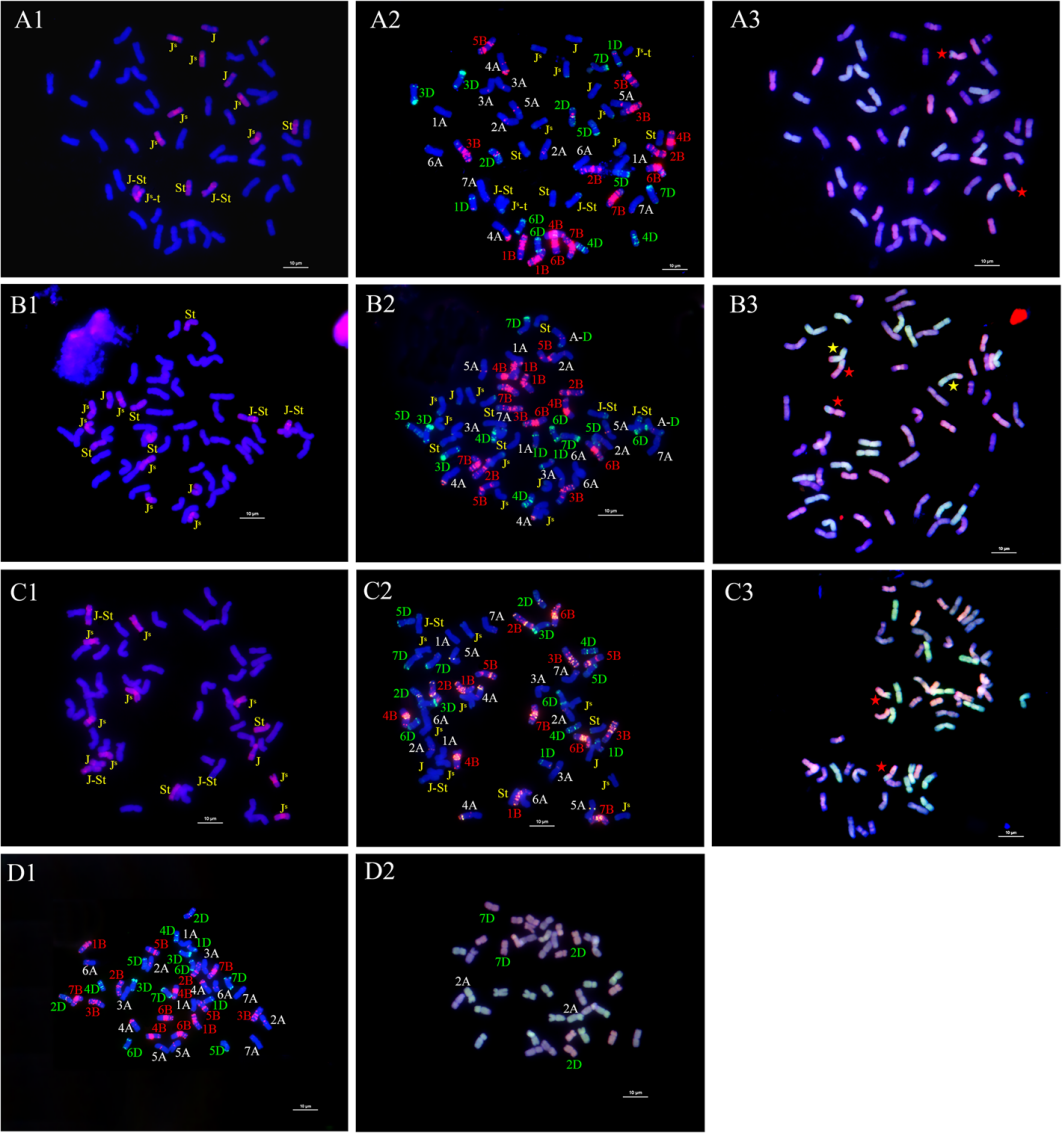
GISH, FISH and McGISH patterns of TE261–1, TE266–1, TE346–1 and YN15. GISH patterns of TE261–1 (A1), TE266–1 (B1) and TE346–1 (C1): St (*Ps*. *strigosa*)-genome DNA labeled with Texas-Red-5-dCTP was used as the probe, and YN15 genome DNA was employed as the block. FISH patterns of TE261–1 (A2), TE266–1 (B2), TE346–1 (C2) and YN15 (D1): red signals represent (GAA)_8_ labeled with 5′ TAMRA, and green signals represent pAs1 labeled with fluorescein-12-dUTP. McGISH patterns of TE261–1 (A3), TE266–1 (B3), TE346–1 (C3) and YN15 (D2): A genomes are labeled with fluorescein-12-dUTP; D genomes are labeled with Texas-Red-5-dCTP; and B-genome DNA (gray) is used for blocking. A-D (7D) translocation are indicated by red asterisks in McGISH patterns (A3, B3 and C3); A-D (2A) translocation are indicated by yellow asterisks in TE266–1 (B3); and 2A, 2D and 7D are marked in the McGISH patterns of YN15 (D2)

**Fig. 2:**
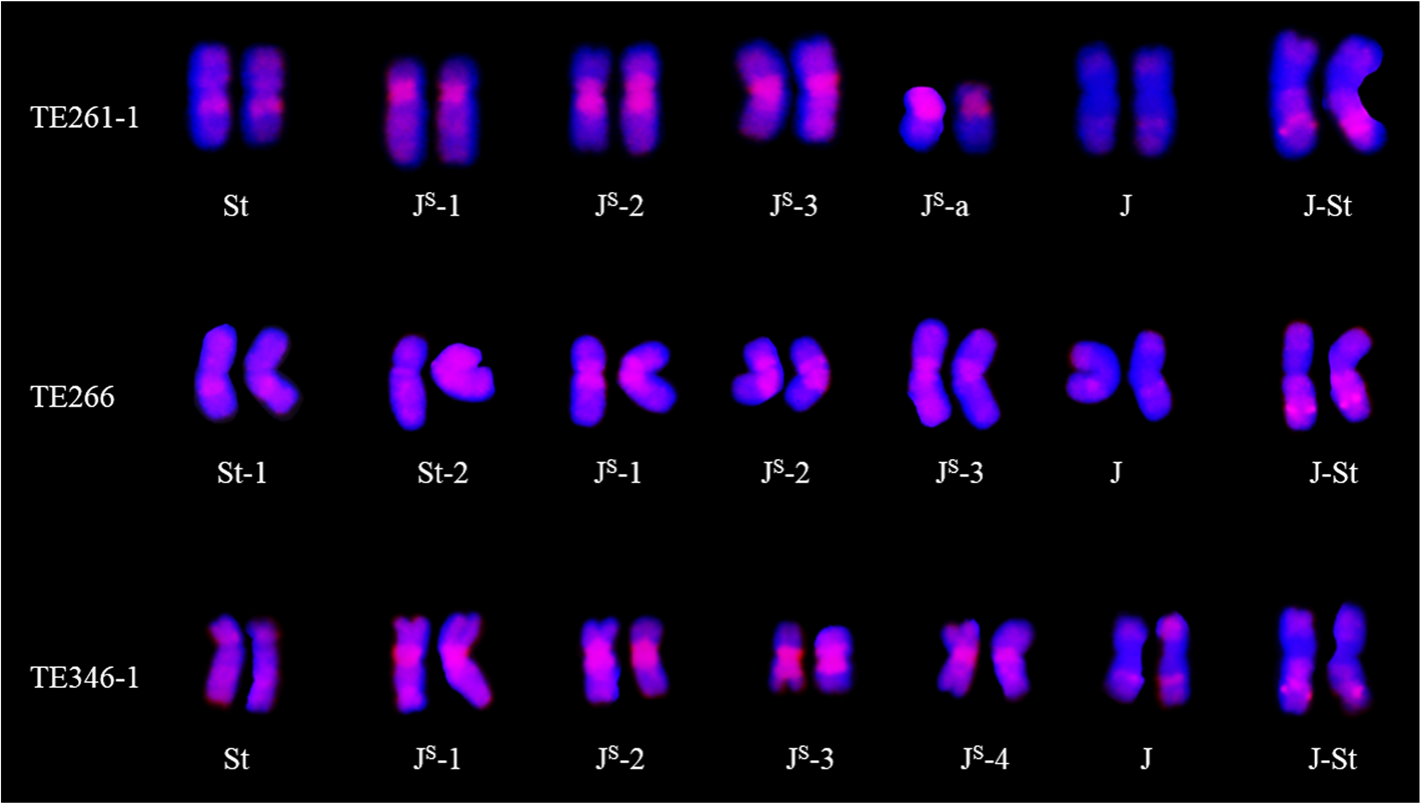
Alien chromosome GISH patterns of the three *Trititrigia* octoploids. St-genome DNA labeled with Texas-Red-5-dCTP was used as the probe, and YN15 genome DNA was employed as the block, J^S^-a represents acrocentric chromosomes from the J^S^ genome

GISH, FISH and McGISH were used to identify the chromosomal constitution of TE261–1, TE266–1 and TE346–1. The GISH (Fig. 1-A1) and FISH (Fig. 1-A2) results indicated that TE261–1 contained 42 wheat chromosomes and 14 *Th. intermedium* chromosomes (Fig. 2), including one pair of **St**-genome chromosomes that were completely labeled with probe signals, one pair of **J**-genome chromosomes labeled only in the telomeres, three pairs of **J**^**S**^-genome chromosomes with obvious labeling in centromere areas, one pair of acrocentric chromosomes from the **J**^**S**^ genome, and one pair of **J-St** translocated chromosomes. The **J**^**S**^ acrocentric chromosomes (Fig. 2, Supplemental Fig. 1-A2), were first discovered in the TE series of *Trititrigia* octoploids.

The results revealed that TE266–1 also contained 42 wheat chromosomes and 14 *Th. intermedium* chromosomes (Fig. 1-B1, B2, B3 and Fig. 2), including two pairs of **St**-genome chromosomes, one pair of **J**-genome chromosomes, three pairs of **J**^**S**^-genome chromosomes, and one pair of **J-St** translocated chromosomes. As shown in Fig. 1-C1, C2, C3 and Fig. 2, TE346–1 also contained 42 wheat chromosomes and 14 *Th. intermedium* chromosomes, including one pair of **St**-genome chromosomes, one pair of **J**-genome chromosomes, four pairs of **J**^**S**^-genome chromosomes and one pair of **J-St** translocated chromosomes.

### Variation in the wheat chromosomes in the three *Trititrigia* octoploids

**Fig. 3:**
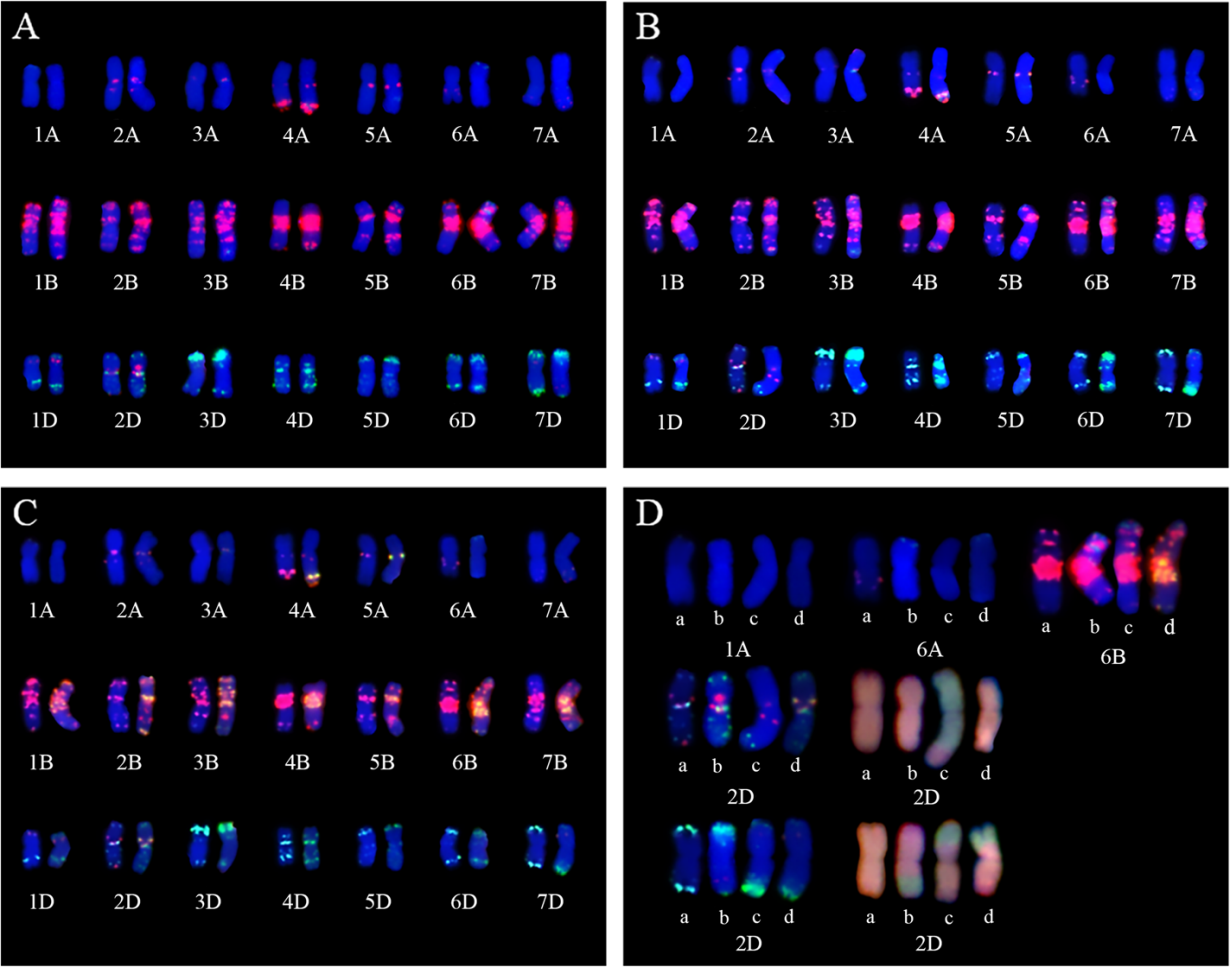
FISH patterns for comparison of YN15 with the three *Trititrigia* octoploids. In the FISH patterns comparing YN15 with TE261–1 (A), TE266–1 (B), and TE346–1 (C), the results for one pair of chromosomes for YN15 are on the left, and those for the *Trititrigia* octoploids are on the right. D: FISH and McGISH patterns for the comparison of YN15 with three *Trititrigia* octoploids on various chromosomes. The patterns in a, b, c and d indicate the corresponding chromosomes of YN15, TE261–1, TE266–1 and TE346–1, respectively. The lower right part of Fig. D shows the McGISH patterns of chromosome 2D and 7D in YN15 and three *Trititrigia* octoploids

FISH analysis were used to identify different wheat chromosomes of TE261–1, TE266–1, TE346–1 and YN15 while using labled (GAA)_8_ (red signals) and pAs1 (green signals) as probes. McGISH analysis were also conducted while using labeled A (green signals) and D (red signals) genomes as probes, B-genome DNA (gray) is used for blocking. The wheat chromosome variations in the three *Trititrigia* octoploids compared with their common wheat parent YN15 based on FISH and McGISH patterns. For the **A**-genome chromosomes, the hybridization signals of pAs1 are not visible on 1**A** in TE261–1, TE266–1 and TE346–1 (Fig. 3D). An absence of (GAA)_8_ signals on 6**A**L and additional pAs1 signals at the 6**A**S subtelomeric position were observed in TE261–1 and TE346–1, while only the absence of (GAA)_8_ signals on 6**A**L was observed in TE266–1 (Fig. 3D).

Compared with YN15, the signals on the 6**B** chromosome in TE261–1, TE266–1 and TE346–1 also changed (Fig. 3A-D). Interspersed green signals for the pAs1 probe were detected on 6**B**S of TE261–1 and TE266–1, and the length of the 6**B**S chromosomes appeared to be extended in TE266–1 and TE346–1. Faint red (GAA)_8_ signals were also observed on the 6**B**L chromosomes in the three *Trititrigia* octoploids.

The signals of the **D**-genome chromosomes in TE261–1, TE266–1 and TE346–1 appeared to vary significantly. FISH and McGISH results indicated that in TE266–1, the 2**D** chromosomes were replaced with one pair of **A-D** translocated chromosomes; the main body of the **A-D** translocated chromosomes was derived from 2**A** chromosomes according to the pAs1 signals, while part of the long arm was replaced with a 2**D** chromosome segment (Fig. 3-B, 3-D).

The FISH results showed that the original strong pAs1 signals on 7**D**S or 7**D**L in YN15 were replaced with faint (GAA)_8_ and pAs1 signals in TE261–1, TE266–1 and TE346–1. The McGISH results indicated that the partial long arm of the 7**D** chromosome in TE261–1 was replaced with the chromosome segment of the **A**-genome chromosome (Fig. 1-A3, Fig. 3-D). However, the translocated **A**-genome chromosome segment was located on the short arm in TE266–1 and TE346–1, not the long arm as in TE261–1. The source of the **A**-genome chromosome segment was uncertain due to the lack of a specific FISH signal in this **A**-genome chromosome segment.

**Fig. 4:**
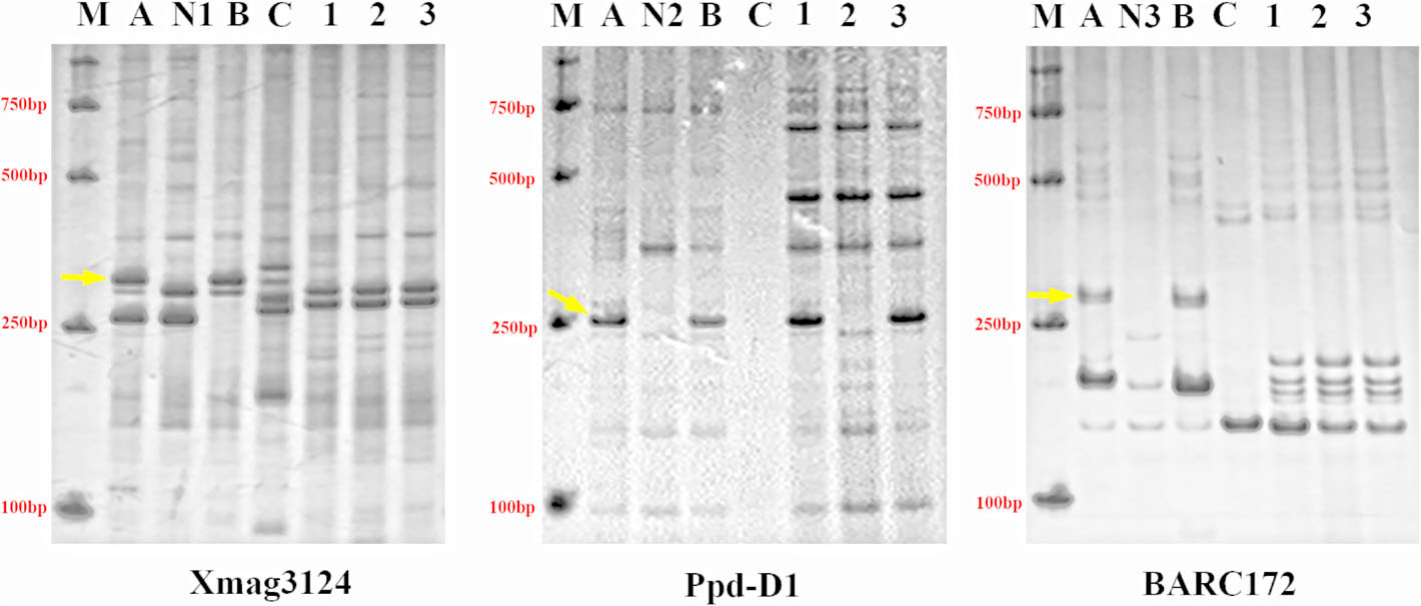
Results of amplification of the wheat chromosome-specific markers *Xmag3124* (1A), *Ppd-D1* (2D) and *BARC172* (7D). M: Marker DL2000, A: CS, N1: CS Nulli-tetrasomes N1AT1D, N2: N2DT2B, N3: N7DT7B, B: YN15, C: *Th. intermedium*, 1: TE261–1, 2: TE266–1, 3: TE346–1

Specific molecular markers of the wheat chromosomes were employed to verify the chromosomal variation and detect the variation type in the three *Trititrigia* octoploids. The specific band (~ 300 bp) of YN15 amplified with the specific marker *Xmag3124*, which was located on 1**A**, was not observed in the three *Trititrigia* octoploids (Fig. 4 and Supplemental Fig. 4). The specific band (~ 150 bp) of YN15 detected by marker *GPW4344*, which was located on 6**A** (Supplemental Fig. 2 and Supplemental Fig. 4), were also absent in the three *Trititrigia* octoploids, and a 6**A** specific band (~ 300 bp) absent in TE346–1 were detected by *GPW7465*. The 6**B** specific band (~ 200 bp) detected by the marker *Xgwm219* showed a 10 bp insertion in three *Trititrigia* octoploids (Supplemental Fig. 2 and Supplemental Fig. 4). The results for several specific markers, such as *Xgdm77*, *Xgdm35*, *Barc095*, *Ppd-D1*, and *Xwmc245*, located on the 2**D** chromosomes (Fig. 4, Supplemental Fig. 2 and Supplemental Fig. 4) demonstrated great variation, i.e., the absent of specific bands in the 2**D** chromosomes in TE266–1, but the 2**D** chromosomes of TE261–1 and TE346–1 remained unchanged. Two specific bands (240, 200 bp) absent in three *Trititrigia* octoploids were detected by *Barc172* and *Barc053* located on the 7**D** chromosomes, (Fig. 4, Supplemental Fig. 2 and Supplemental Fig. 4), respectively.

The sequences of the primers used for these 16 chromosome-specific markers were employed to run local BLAST searches against the database of the *T. aestivum* (Chinese Spring, CS) whole-genome sequence. Eleven markers were screened according to screening criteria including the appropriate chromosomes, initial position, terminal position and degree of strict matching (Supplemental Table 2). For example, the chromosomes mapped by BLAST should be matched the specific chromosomes detected by PCR; the higher matching degree means the better option, and the 3′ of primer must be matched; the absolute value of margin between forward primer initial position and reverse primer terminal, should match the size of specific bands; and last, the appropriate position mapped by the markers were picked out. Compared BLAST with PCR results after screening, the size of specific bands amplified with majority markers were similar (Fig. 4), but the 7**D** specific band (~ 240 bp) detected by *Barc053* was different from BLAST result (295 bp), and difference exist in Ppd-D1 (R1) result of PCR (250 bp) and BLAST (413 bp). The difference could be attributed to the sequence diversity between CS and YN15. The screened markers were then labeled at the specific location of the corresponding chromosomes using MapGene2Chromosomes v2, according to their physical position on the chromosomes (Supplemental Fig. 3). The positions of the variations detected with markers were consistent with the FISH signals on the 6**A**, 6**B**L, 2**D** and 7**D** chromosomes, while the variation at the 1**A**L telomere was detected with markers, rather than FISH signals.

### Phenotypic evaluation of the three *Trititrigia* octoploids

The reactions to stripe rust, powdery mildew and aphids in TE261–1, TE266–1 and TE346–1 were evaluated at the seedling and adult plant stages using the wheat parents YN15 and *Th*. *intermedium* as controls (Table 1). At the seedling stage, TE261–1, TE266–1, and TE346–1 were immune to the stripe rust race CYR32 and resistant to the powdery mildew race E09. At the adult stage, all three *Trititrigia* octoploids showed good resistance to stripe rust and powdery mildew in the field. TE261–1 and TE346–1 also showed moderate resistance to aphids at the adult stage, while TE266–1 and YN15 were susceptible. As the common wheat parent YN15 is susceptible to stripe rust, powdery mildew and aphids, whereas *Th*. *intermedium* is immune to all three, we deduce that the resistance of *Trititrigia* is derived from *Th*. *intermedium*.

**Table 1:** Evaluation of stripe rust, powdery mildew and aphid resistances in TE261–1, TE266–1 and TE346–1

Materials	Seedling stage		Adult plant stage		
	Stripe rust CYR32	Powdery mildew E09	Stripe rust	Powdery mildew	Aphid
*Th*. *intermedium*	0	0	0	0	0
YN15	4	4	4	4	5
TE261–1	0	2	0;	0;	2
TE266–1	0	1	0;	0;	4
TE346–1	0	1	0;	0;	3

## Discussion

*Th. intermedium* is a valuable perennial species of Triticeae for wheat improvement due to its resistance to a number of wheat diseases and pests as well as its stress tolerance and high crossability with various *Triticum* species [8–11, 45]. Partial wheat-*Th. intermedium* amphiploids showing high cross-compatibility with wheat are desirable ‘bridge’ materials for transferring desirable genes from *Th. intermedium* to common wheat [19, 20].

Chromosome counting in metaphase spreads revealed that the chromosome number of these three octoploids of *Trititrigia* was consistently 2n = 56. Molecular cytogenetic analysis then revealed that the three *Trititrigia* accessions all carry 42 wheat chromosomes and 14 *Th. intermedium* chromosomes and that the 14 alien (*Th. intermedium*) chromosomes are composed of a mixed genome consisting of **J**-, **J**^**S**^- and **St**-genome chromosomes rather than a single **J**, **J**^**S**^ or **St** genome, similar to other reported of *Trititrigia* octoploids [21, 46, 47]. The mechanism underlying the formation of the mixed genome is uncertain, and with development of new techniques and methods, such as oligo-FISH painting system [48] further research will contribute to elucidating the homologous relationship and integrality between the **J**, **J**^**S**^ and **St** genomes and the origin and evolution of *Th. intermedium*.

Two types of structural variation in the alien chromosomes were detected: **J-St** translocated chromosomes, which were present in all three *Trititrigia* octoploids, and **J**^**S**^ acrocentric chromosomes in TE261–1 (Fig. 2), which might have formed via the centromere breakage of one pair of **J**^**S**^-genome chromosomes. **J-St** translocated chromosomes also exist in many TE series of *Trititrigia* octoploids [21, 26, 28], but **J**^**S**^ acrocentric chromosomes were first discovered in the TE series of *Trititrigia* octoploids. The formation of these two types of variations might occur through chromosome breakage and fusion when F_1_ hybrids obtained through distant hybridization are backcrossed with hexaploid wheat [34].

Although the alien chromosomal constitution of three *Trititrigia* octoploids was different, some of the alien chromosomes were probably identical according to the GISH and FISH signals. For example, the **J**-genome chromosomes with the satellite sequence and the **J-St** translocated chromosomes in these three *Trititrigia* octoploids were similar. The **St** chromosomes of TE261–1 were also similar to the **St**-1 chromosomes of TE266–1, and the **St** chromosomes of TE346–1 were similar to the **St**-2 chromosomes of TE266–1 (Fig. 2).

In addition to the structural variation in the alien chromosomes, structural variations, such as the variations detected in the 1**A**, 6**A**, 6**B**, 2**D** and 7**D** chromosomes, occurred in the wheat chromosomes as well. The results indicated that during the formation of partial amphiploids, the wheat chromosomes underwent various types of chromosomal recombination due to chromosome segments introgressed from *Th. intermedium* into the common wheat chromosomes, although the introgressed segments were too small to detect via GISH. The structural variations may have been generated via recombination between different wheat chromosomes, such as homoeologous chromosomal recombination between **A**-, **B**-, and **D**-genome chromosomes, influenced by the *Th. intermedium* chromosomes. The variation in chromosomal patterns of repetitive sequences (e.g. chromosome 6**B**) could be also caused by repetitive sequence reorganization (amplification/loss). After hybridization, genomes can undergo genetic and epigenetic changes (genomic shock) which can reorganize their structure.

Similar chromosomal variations were detected in the 1**A** and 6**A** chromosomes of the three *Trititrigia* and might have been derived from the same origin in the earlier generations of backcrosses. Additionally, there were distinct variations in the 6**B**, 2**D** and 7**D** chromosomes of these *Trititrigia* octoploids, such as pAs1 signals at different positions of 6**B**S in TE266–1 and TE346–1 and the 2**A**-2**D** translocation in TE266–1. The variations in the 7**D** chromosomes of the three *Trititrigia* octoploids were also different. McGISH indicated that the partial 7**D**S chromosomes of TE266–1 and TE346–1 were replaced with an **A**-genome chromosome segment, but the corresponding position in TE261–1 was on the 7**D**L chromosome. The original position replaced in 7**D**L or 7**D**S was difficult to discern via FISH because the signals on the two arms of the 7**D** chromosomes were similar. The results of molecular marker analyses also supported the results of FISH and McGISH in TE261–1. Additional work will be needed to obtain strong evidence indicating how these chromosomal recombinations occurred as well as the effect of these chromosomal variations.

The drastic and abundant chromosome variations were detected in these three *Trititrigia* octoploids, a kind of generic hybrid between wheat and *Th. intermedium*. However, we guess the more drastic variations might exist in early-generation of *Trititrigia* octoploids, and then backcrosses with YN15 resulted in reducing variability and increasing stability. We hypothesized that *Th. intermedium*, as a natural generic hybrid, possessed drastic chromosomal variation causing difficulty to confirm the progenitor diploid species.

Although FISH results using general probes consisting of (GAA)_8_ and pAs1 were more abundant, colorful and characteristic to distinguish most common wheat chromosomes, it was still insufficient to identify the alien chromosomes in present amphiploids. Li et al. had reported an efficient Oligo-FISH painting system for revealing chromosome rearrangements and polyploidization in Triticeae [48], while using novel comparative genome-based oligo painting FISH procedure. These would be beneficial and helpful for determining the alien *Thinopyrum* chromosomes in these amphiploids in the future studies.

Phenotypic evaluation indicated the successful transfer of stripe rust and powdery mildew resistance from *Th. intermedium* to these three *Trititrigia* octoploids and that TE261–1 also exhibited moderate resistance to aphids. In addition, the chromosomal constitution of TE261–1, TE266–1 and TE346–1 is different from that of other TE series of *Trititrigia* octoploids reported previously [21, 26, 28]. Therefore, these accessions could be employed as bridge parents for crossing with wheat to develop addition, substitution or translocation lines to generate new rust-, powdery mildew- and aphid-resistant germplasms for wheat breeding.

## Conclusions

Three octoploid *Trititrigia* accessions (TE261–1, TE266–1, and TE346–1) with good resistances to diseases and aphids were characterized by GISH, FISH, McGISH and molecular marker analyses. Molecular cytogenetic analysis revealed that these *Trititrigia* accessions all carry 42 wheat chromosomes and 14 *Th. intermedium* chromosomes, and the 14 alien (*Th. intermedium*) chromosomes are composed of a mixed genome consisting of **J**-, **J**^**S**^- and **St**-genome chromosomes rather than a single **J**, **J**^**S**^ or **St** genome. Different structural variations occurred in the wheat chromosomes, such as the variations detected in the 1**A**, 6**A**, 6**B**, 2**D** and 7**D** chromosomes as well. Results indicated that the wheat-*Th. intermedium* partial amphiploids possess 14 alien chromosomes which belong to a mixed genome consisting of J-, J^S^- and St- chromosomes, and 42 wheat chromosomes with different structural variations.

## Methods

### Plant materials

Three *Trititrigia* octoploid accessions (TE261–1, TE266–1, and TE346–1) were selected from BC_1_F_8_ of common wheat Yannong15 crossed with *Th. intermedium*, and selfed for some generations. *Th. intermedium* from the original accession at the Northwest Institute of Botany, Chinese Academy of Sciences, was provided by the academician Li ZS; *Pseudoroegneria strigose*, *Triticum urartu* Thum. ex Gandil. (2n = 2x = 14, **AA**), *Aegilops speltoides* Tausch. (2n = 2x = 14, **BB**), *Aegilops tauschii* Coss. (2n = 2x = 14, **DD**) and the CS nulli-tetrasomic accessions N1AT1B, N6AT6B, N2DT2A, N7DT7A were provided by the researcher Li LH from the Institute of Crop Sciences, Chinese Academy of Agricultural Sciences.

### DNA extraction, PCR amplification and marker analysis

The CTAB method was used to extract total genomic DNA from the tender leaves [49] of *Pseudoroegneria strigosa* (**St** genome), *Triticum urartu* (**A** genome), *Aegilops speltoides* (**B** genome) and *Aegilops tauschii* (**D** genome). In total, 176 pairs of SSR primers [50, 51] (https://wheat.pw.usda.gov) were used to identify chromosomal variations. PCR amplification was performed according to Beales et al. [52], and marker screening was conducted by means of polyacrylamide gel electrophoresis (PAGE). The selected markers were analyzed with BLAST (Basic Local Alignment Search Tool) based on the database containing the *Triticum aestivum* (CS) whole genome sequence (IWGSC RefSeq v1.0, https://wheat-urgi.versailles.inra.fr/morgoth/Seq-Repository/BLAST) and then labeled on the corresponding chromosomes using MapGene2Chromosomes v2 (http://mg2c.iask.in/mg2c_v2.0/).

### Chromosome slide preparation, meiotic preparation, GISH, FISH and McGISH

Fresh root tips were collected from germinating seeds treated with nitrous oxide (N_2_O) for 2 h [53] and then immersed in 90% glacial acetic acid to fix cell division. The preparation of root tip cell chromosome slides and analysis was performed referring to the method of Han [46]. When the flag leaf of wheat was spread, young spikes were sampled and anthers at metaphase I (MI) of meiosis were fixed in Carnoy’s solution. The meiotic chromosomes were prepared from pollen mother cells (PMCs) by knocking and pressing anthers in 45% acetic acid, after which the chromosome configuration in PMC metaphase I (MI) was observed.

**St**-genome DNA and **D**-genome DNA were labeled with Texas-Red-5-dCTP. The clone pAs1 (GenBank: D30736.1) is a repetitive DNA sequence (1015 bp) from *Aegilops tauschii* [37, 54]. The pAsl clone and **A**-genome DNA were labeled with fluorescein-12-dUTP using the nick translation method. Oligonucleotides (GAA)_8_ with 5′ TAMRA were synthesized by Sangon Biotech (Shanghai, China). The procedures for GISH, FISH and McGISH; signal detection; and image collection were done according to Han et al. [55] and Cui et al. [56].

The reaction volume for in situ hybridization was 10 μL per slide. GISH was conducted using a probe of 50 ng **St**-genome DNA, with a block of 6000 ng YN15 DNA; FISH was performed with 5 ng (GAA)_8_ and 200 ng pAs1; and McGISH was carried out with probes of 50 ng A-genome DNA and 100 ng **D**-genome DNA and a block of 8000 ng **B**-genome DNA diluted to the desired total volume with ddH_2_O. The in situ hybridization program consisted of denaturation at 95 °C for 5 min and hybridization at 55 °C for 6 h. The fluorescent signals of FITC (Excitation Filter EX465–495, Dichroic Mirror DM505, Barrier Filter BA515–555), TRITC (Excitation Filter EX540/25, Dichroic Mirror DM565, Barrier Filter BA605/55) and DAPI (Excitation Filter EX340–380, Dichroic Mirror DM400, Barrier Filter BA435–485) were detected, and images were collected using a NIKON eclipse Ni-U fluorescence microscope; the images were processed using NIS-Elements BR 4.00.12 software. After rinsing with 2X SSC solution, the slides were used for in situ hybridization again.

### Evaluation of resistance to powdery mildew, stripe rust and aphids

The powdery mildew and stripe rust resistance of plants in the seedling stage were tested in a plant incubator. The wheat variety Huixianhong was employed for inoculation of the *Blumeria graminis f. sp. tritici* (*Bgt*) pathotype E09 and the *Puccinia striiformis f. sp. tritici* (*Pst*) pathotype CYR32. Thirty seeds of the test materials were grown in seedling plates and placed in a plant incubator under 12 h light (25,000 lx) at 25 °C and 12 h of darkness at 17 °C, with 70% relative humidity. Huixianhong and YN15 were used as controls. Inoculation with E09 and CYR32 was achieved by smearing at the one-leaf stage, and the gradient of infection was investigated when Huixianhong had been completely infected; these experiments were repeated three times. The powdery mildew and stripe rust resistances of adult-stage plants were tested via natural infection in an open field. The resistance evaluation criteria for powdery mildew and stripe rust followed the methods of Sheng [57] and Li et al. [58]. In the scale of powdery mildew resistance, 0 is immune; 0; is nearly immune with necrotic flecks; 1 is highly resistant with small lesions (< 1 mm) and thin mycelium; 2 is moderately resistant with small lesions (< 1 mm) and a slightly thick mycelium; 3 is moderately susceptible with lesions (> 1 mm) and a thick mycelium; and 4 is highly susceptible with lesions (> 1 mm) and a consecutive thick mycelium. In the scale of stripe rust resistance, 0 is immune; 0; is nearly immune with tiny flecks; 1 is highly resistant with yellowish-white flecks; 2 is moderately resistant with necrotic plaques around small spore stripes; 3 is moderately susceptible with fading around spore stripes; and 4 is highly susceptible with large spore stripes.

For the identification of aphid resistance, natural aphids were employed, and the evaluation was performed during the wheat grain-filling stage. The test materials were sown on two 1.4-m-long rows with YN15 as CK, *Th. intermedium* planting in pot were put nearby to evaluate the aphid resistance. The investigation of aphid injury and the classification criteria for aphid resistance followed the method of Liu et al. [59]. The number of aphids on 10 seriously aphid-damaged stems was determined for all materials; then, using this number as the numerator and the average number of aphids on YN15 as the denominator, the ratio was computed as the evaluation criterion: 0 is immune with no aphids; 1 is highly resistant with a ratio between 0.01 and 0.30; 2 is moderately resistant with a ratio between 0.31 and 0.6; 3 is slightly resistant with a ratio between 0.61 and 0.90; 4 is slightly susceptible with a ratio between 0.91 and 1.2; 5 is moderately susceptible with a ratio between 1.21 and 1.5; and 6 is highly susceptible, with a ratio > 1.5.

## Supplementary Information

**Additional file 1: Supplemental Fig. 1.** Chromosomal configuration and GISH results for PMC MI in TE261–1, TE266–1 and TE346–1.

**Additional file 2: Supplemental Fig. 2.** Results of the amplification of wheat chromosome-specific markers.

**Additional file 3: Supplemental Fig. 3.** Specific molecular marker map indicating chromosomal variation; chr indicates the corresponding chromosome.

**Additional file 4: Supplemental Fig. 4.** Uncropped images of markers in Fig. 4 and Supplemental Fig. 2.

**Additional file 5: Supplemental Table 1.** Chromosomal configurations of TE261–1, TE266 and TE346–1 at PMC MI.

**Additional file 6: Supplemental Table 2.** Sequence alignment results for the screened wheat chromosome-specific molecular markers.

## Data Availability

The datasets used and/or analysed during the current study are available from the corresponding author on reasonable request.

## Abbreviations

CS: Chinese Spring
FISH: Fluorescence in situ hybridization
GISH: Genomic in situ hybridization
McGISH: Multicolor GISH
YN15: Yannong 15
