# Supplementary material for: Characterization of chromosome constitution in three wheat - Thinopyrum intermedium amphiploids revealed frequent rearrangement of alien and wheat chromosomes
Source: BMC Plant Biol. 2021 Mar 4;21:129. doi: 10.1186/s12870-021-02896-9 (PMC7931331; doi:10.1186/s12870-021-02896-9)
Supplement: Supplementary file 1 — Additional file 1: Supplemental Fig. 1. Chromosomal configuration and GISH results for PMC MI in TE261–1, TE266–1 and TE346–1. [file 12870_2021_2896_MOESM1_ESM.docx]

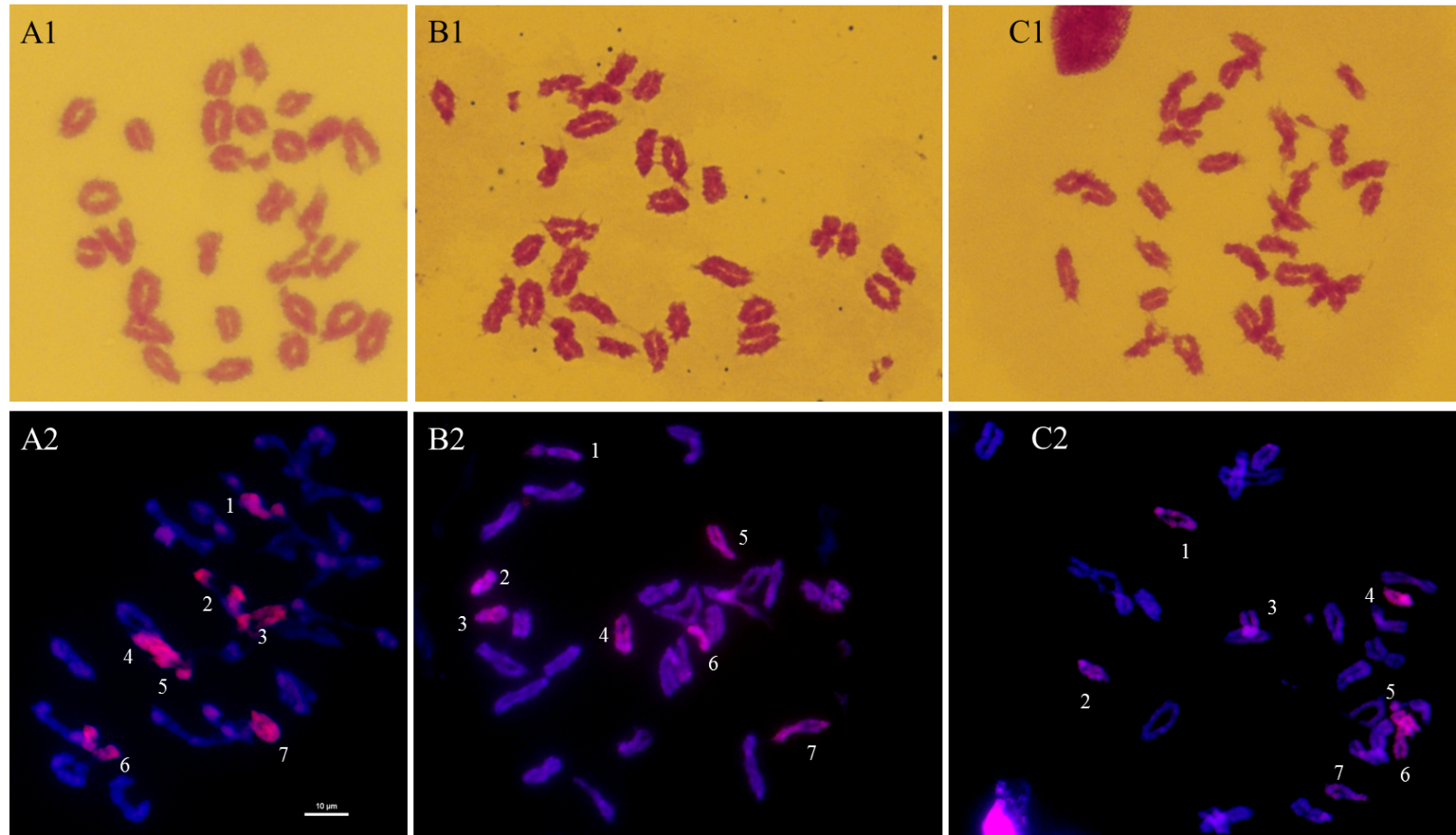


Supplemental Fig. 1. Chromosome configuration of PMC MI in TE261-1 (A1), TE266-1 (B1) and TE346-1 (C1), chromosomes were dyed red by carbol fuchsin, Based on investigating 40 cells in every material, the chromosome configurations of TE261-1, TE266-1, TE346-1 were both 2n=28II=56. GISH of PMC MI in TE261-1 (A2), TE266-1 (B2) and TE346-1 (C2), St (*Ps. Strigosa*) genome DNA labeled with Texas-Red-5-dCTP were used as probe and YN15 genome DNA were used as block, 14 alien chromosomes matched 7II. No. 5 bivalent in Fig. A2 probably formed by the J^S^ acrocentric chromosomes in TE261-1.
