# Supplementary material for: Characterization of chromosome constitution in three wheat - Thinopyrum intermedium amphiploids revealed frequent rearrangement of alien and wheat chromosomes
Source: BMC Plant Biol. 2021 Mar 4;21:129. doi: 10.1186/s12870-021-02896-9 (PMC7931331; doi:10.1186/s12870-021-02896-9)
Supplement: Supplementary file 2 — Additional file 2: Supplemental Fig. 2. Results of the amplification of wheat chromosome-specific markers. [file 12870_2021_2896_MOESM2_ESM.docx]

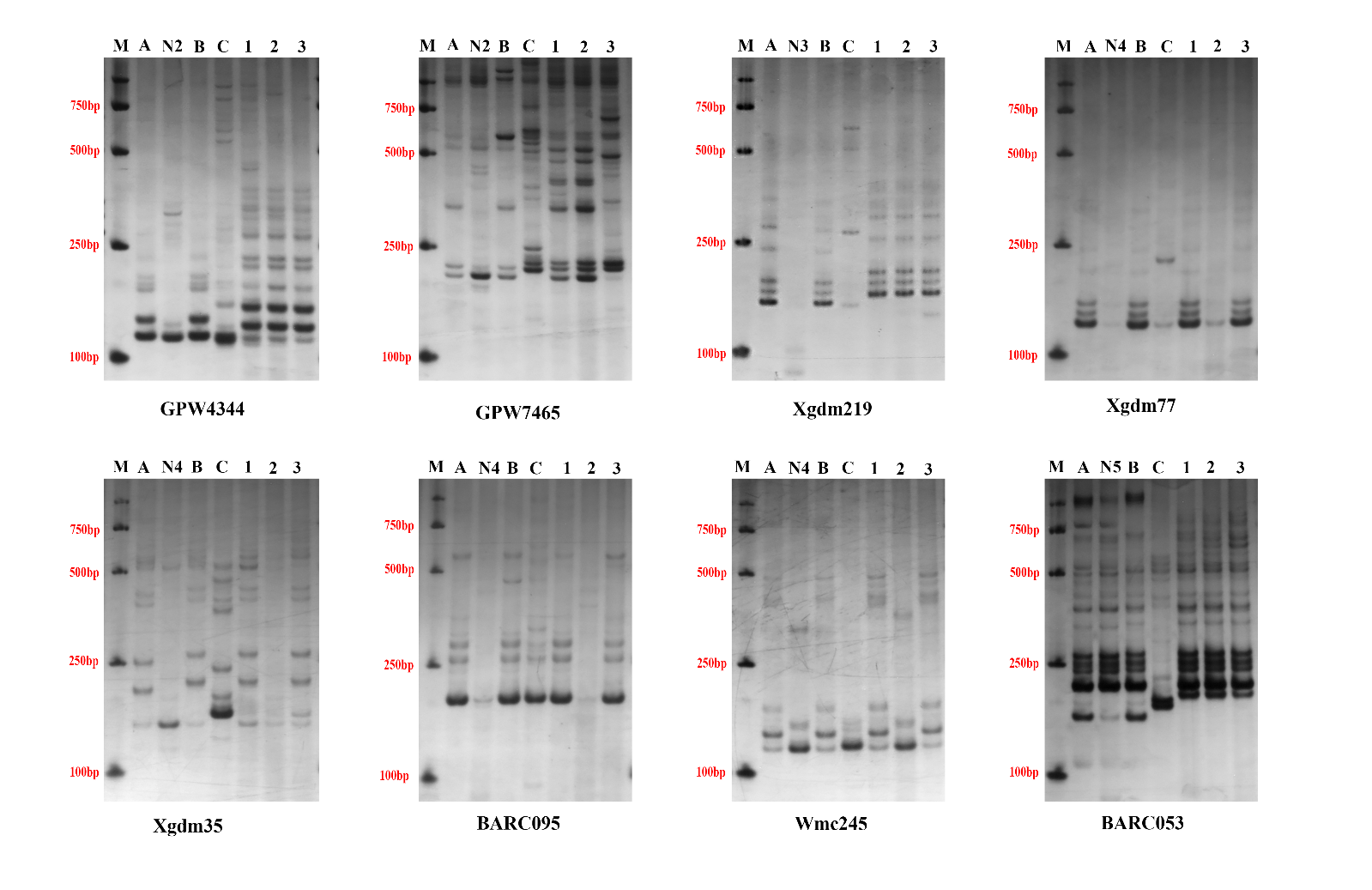


**Supplemental Fig. 2.** Amplified results of wheat chromosome-specific markers. GPW4344, GPW7465 (6A), Xgwm219 (6B), Xgdm77, Xgdm35, BARC095, Wmc245 (2D) and BARC053 (7D).

M. Marker, A. Chinese spring, N1. N1AT1D, N2. N6AT6D, N3. N6BT6D, N4. N2DT2B,

N5. N7DT7B, B. Yannong15, C. *Th*. *intermedium*, 1. TE261-1, 2. TE266, 3. TE346
