# Supplementary material for: Characterization of chromosome constitution in three wheat - Thinopyrum intermedium amphiploids revealed frequent rearrangement of alien and wheat chromosomes
Source: BMC Plant Biol. 2021 Mar 4;21:129. doi: 10.1186/s12870-021-02896-9 (PMC7931331; doi:10.1186/s12870-021-02896-9)
Supplement: Supplementary file 3 — Additional file 3: Supplemental Fig. 3. Specific molecular marker map indicating chromosomal variation; chr indicates the corresponding chromosome. [file 12870_2021_2896_MOESM3_ESM.docx]

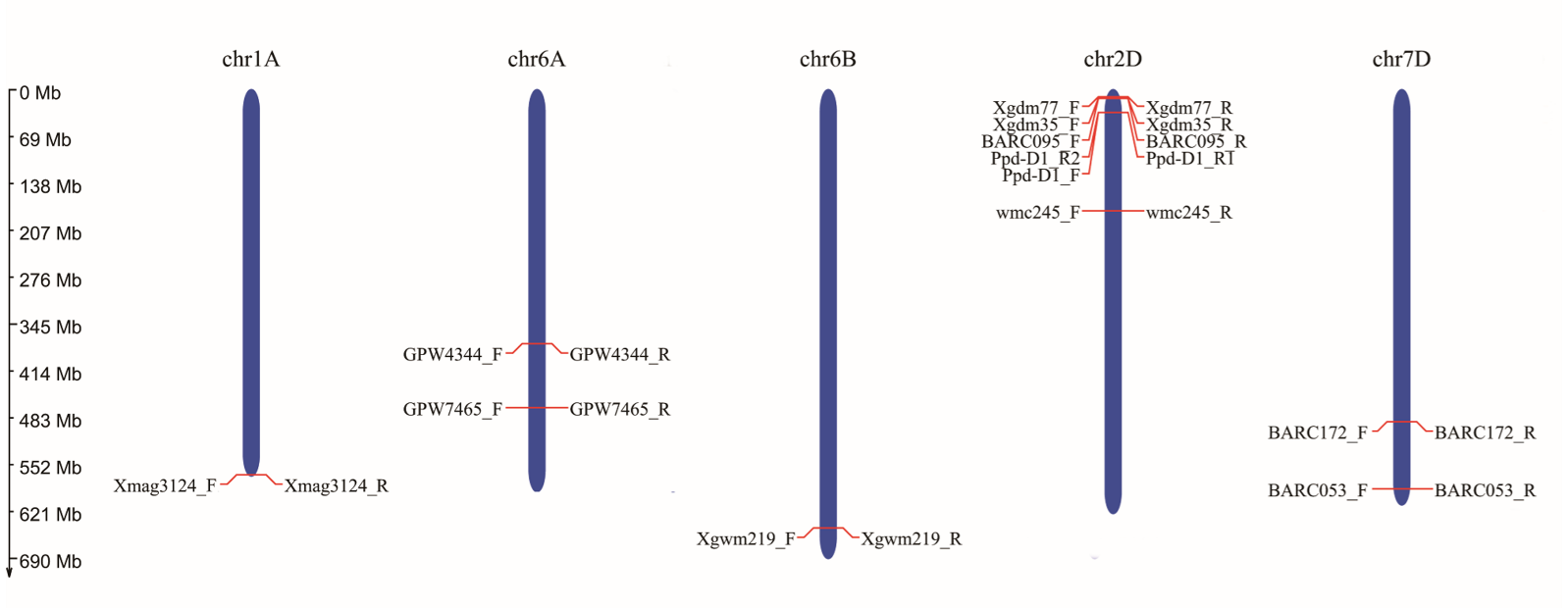


**Supplemental Fig. 3.** Specific molecular marker map indicating chromosomal variation; chr indicates the corresponding chromosome.

The length of the chromosome reflects chromosome size; and positional variation was labeled with molecular markers.
