# Supplementary material for: Characterization of chromosome constitution in three wheat - Thinopyrum intermedium amphiploids revealed frequent rearrangement of alien and wheat chromosomes
Source: BMC Plant Biol. 2021 Mar 4;21:129. doi: 10.1186/s12870-021-02896-9 (PMC7931331; doi:10.1186/s12870-021-02896-9)
Supplement: Supplementary file 4 — Additional file 4: Supplemental Fig. 4. Uncropped images of markers in Fig. 4 and Supplemental Fig. 2. [file 12870_2021_2896_MOESM4_ESM.pptx]

## Slide 1
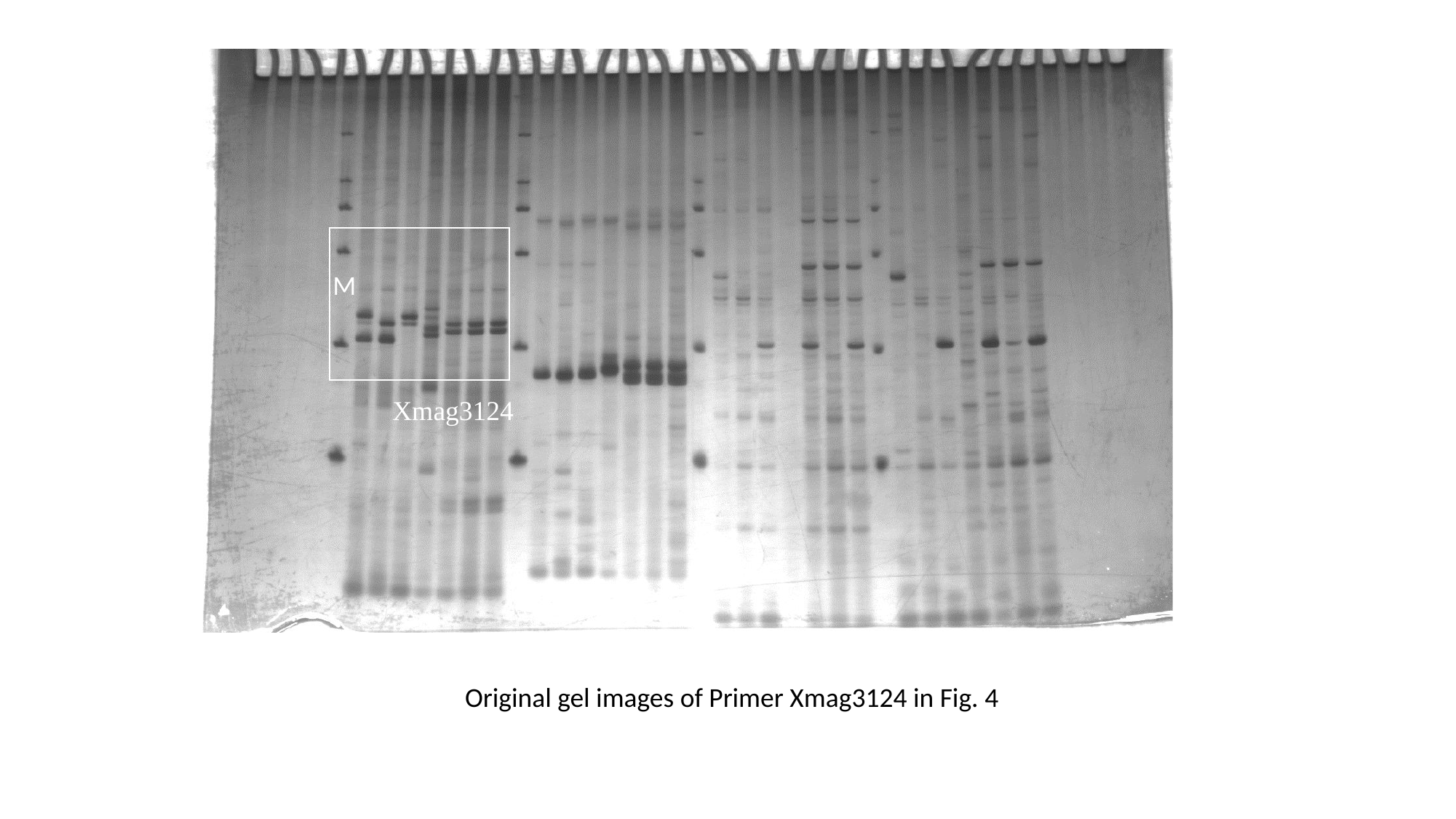

M
Xmag3124
Original gel images of Primer Xmag3124 in Fig. 4

## Slide 2
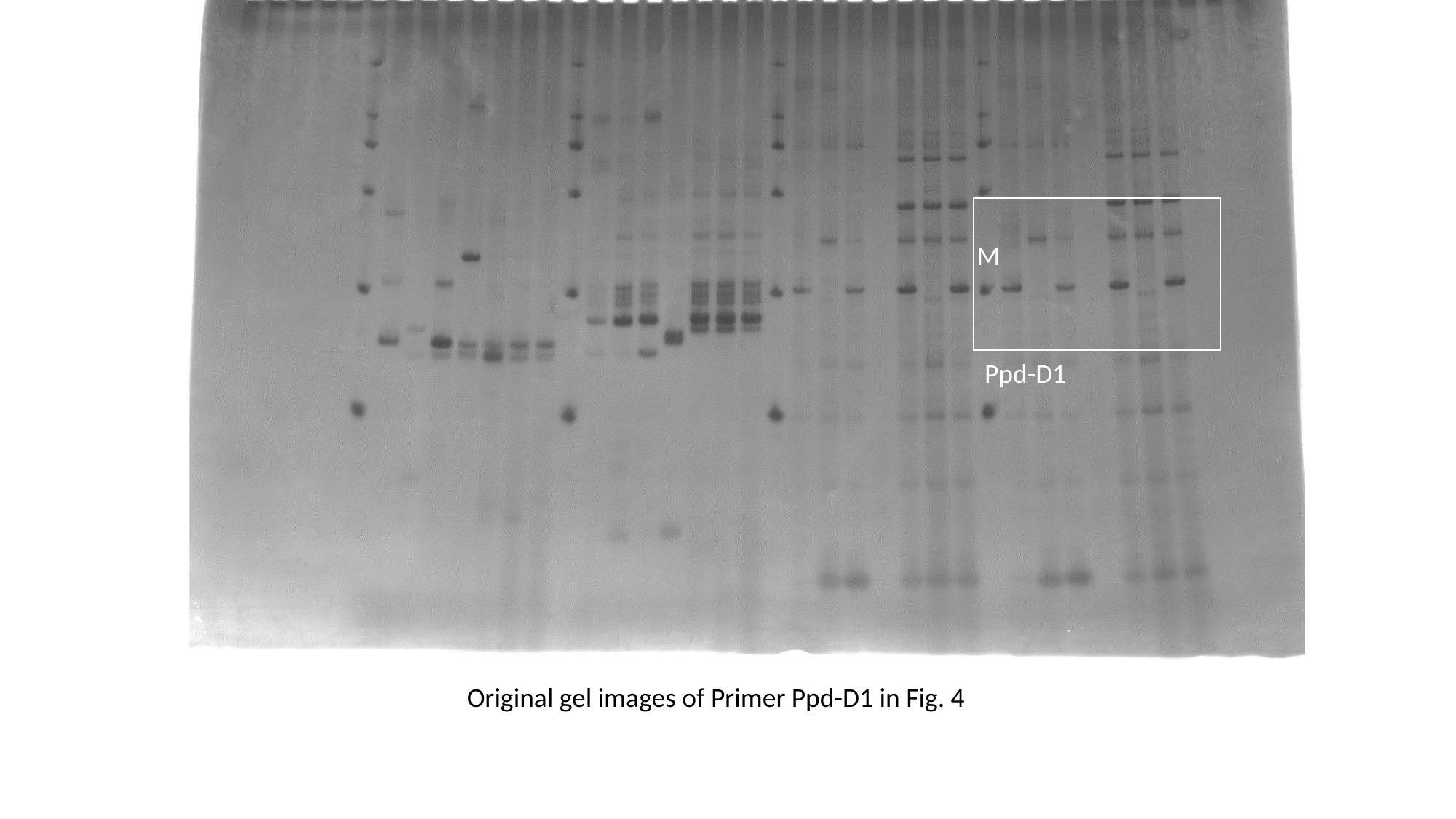

M
Ppd-D1
Original gel images of Primer Ppd-D1 in Fig. 4

## Slide 3
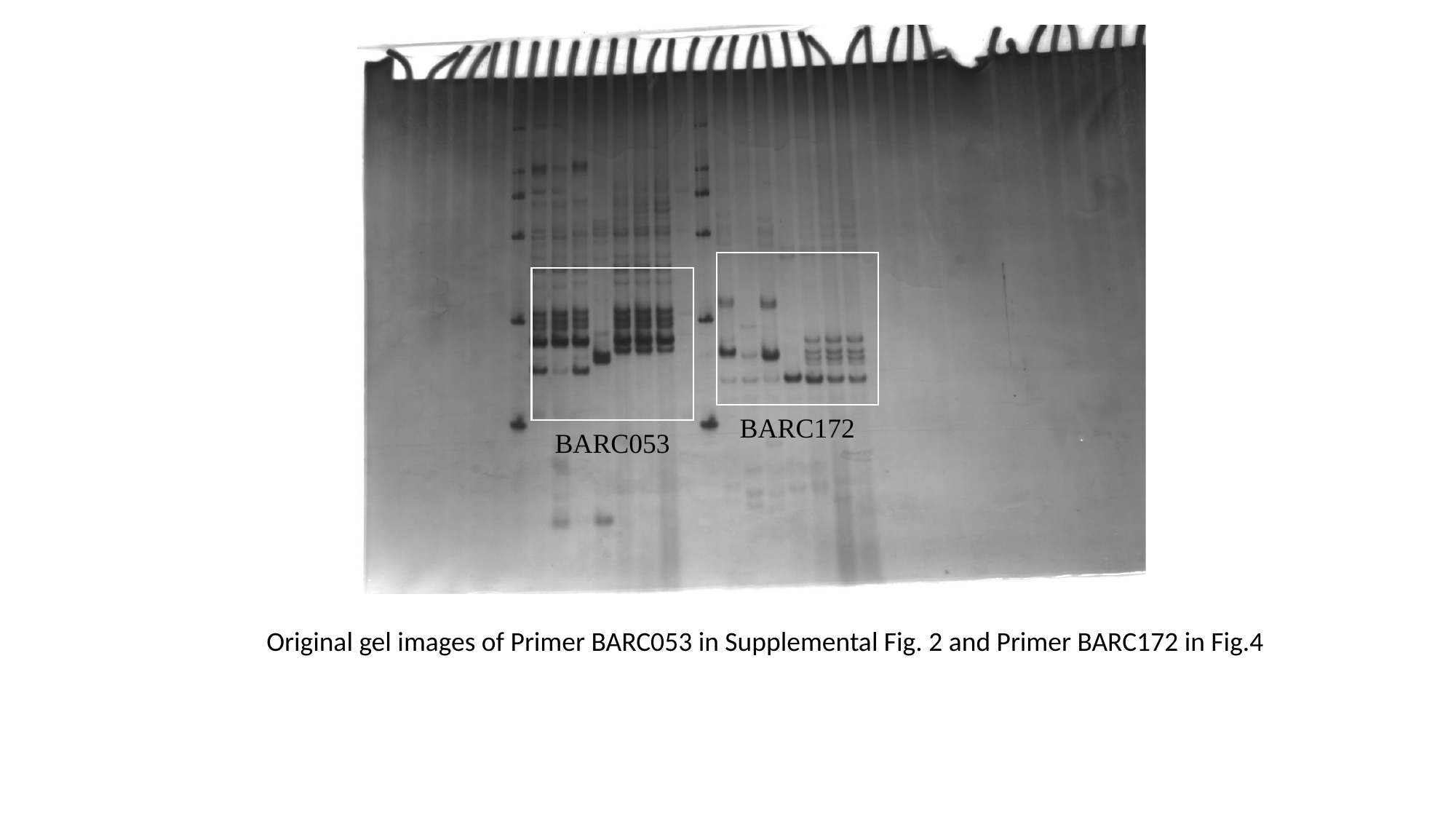

BARC172
BARC053
Original gel images of Primer BARC053 in Supplemental Fig. 2 and Primer BARC172 in Fig.4

## Slide 4
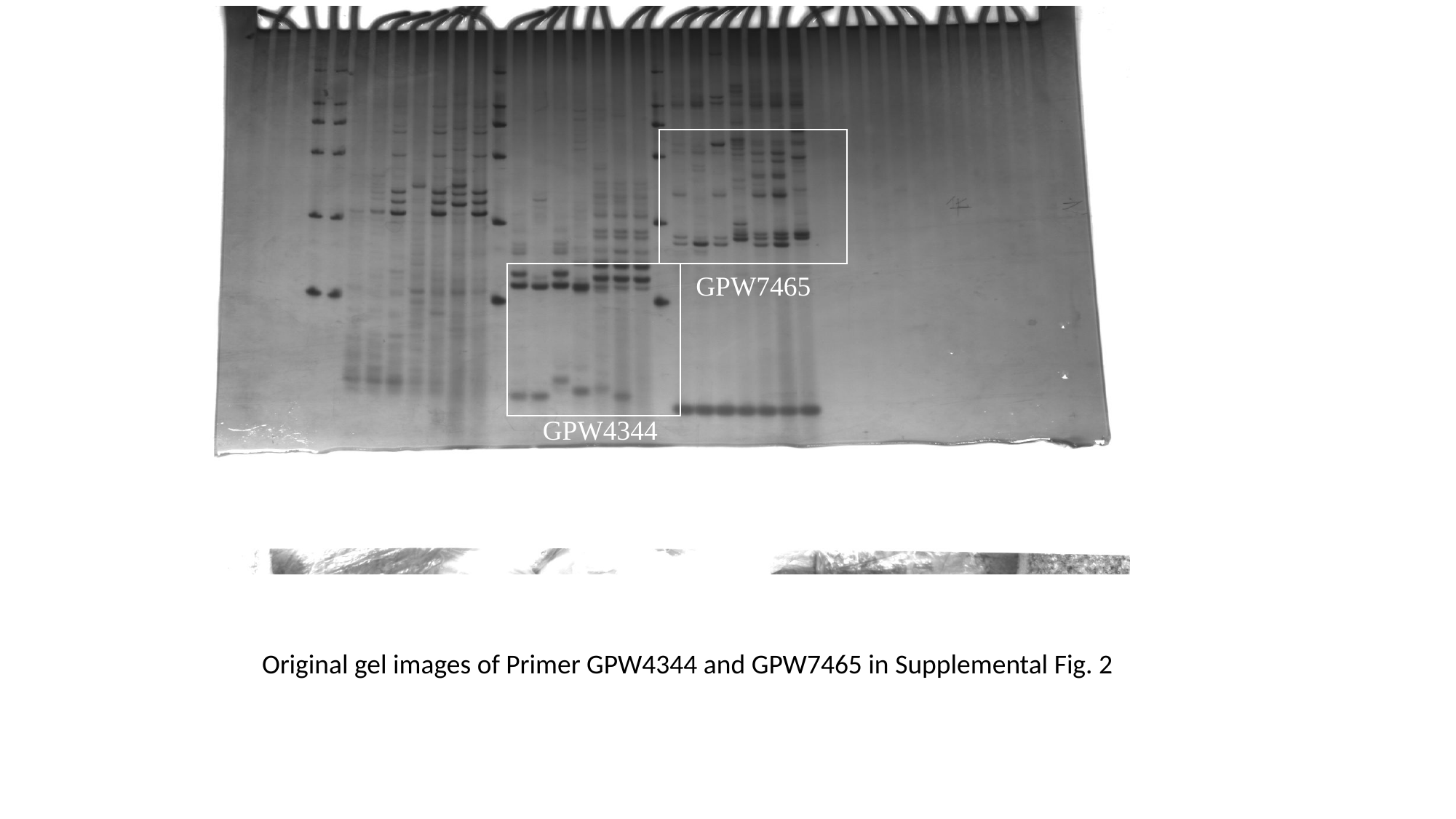

GPW7465
GPW4344
Original gel images of Primer GPW4344 and GPW7465 in Supplemental Fig. 2

## Slide 5
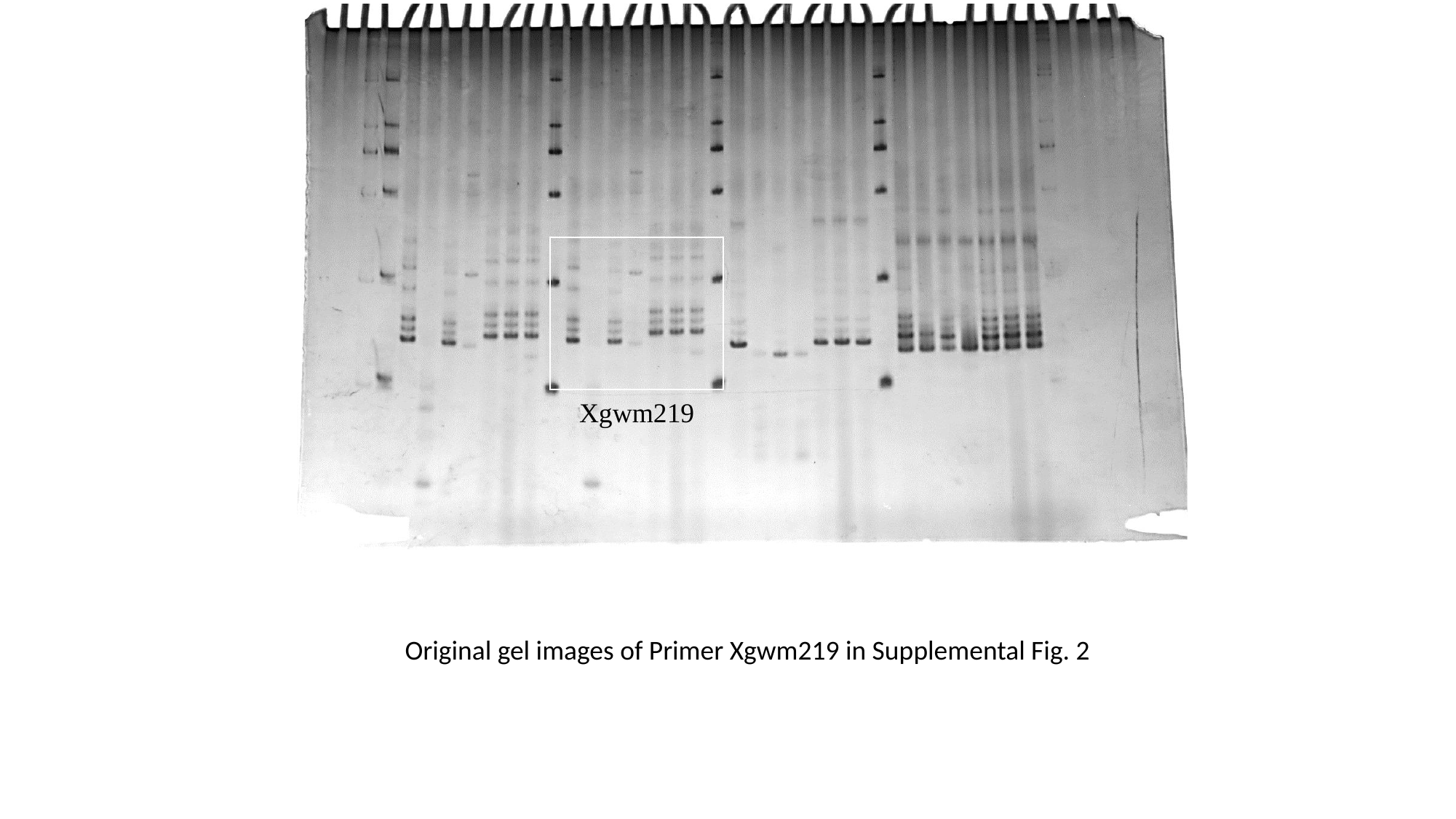

Xgwm219
Original gel images of Primer Xgwm219 in Supplemental Fig. 2

## Slide 6
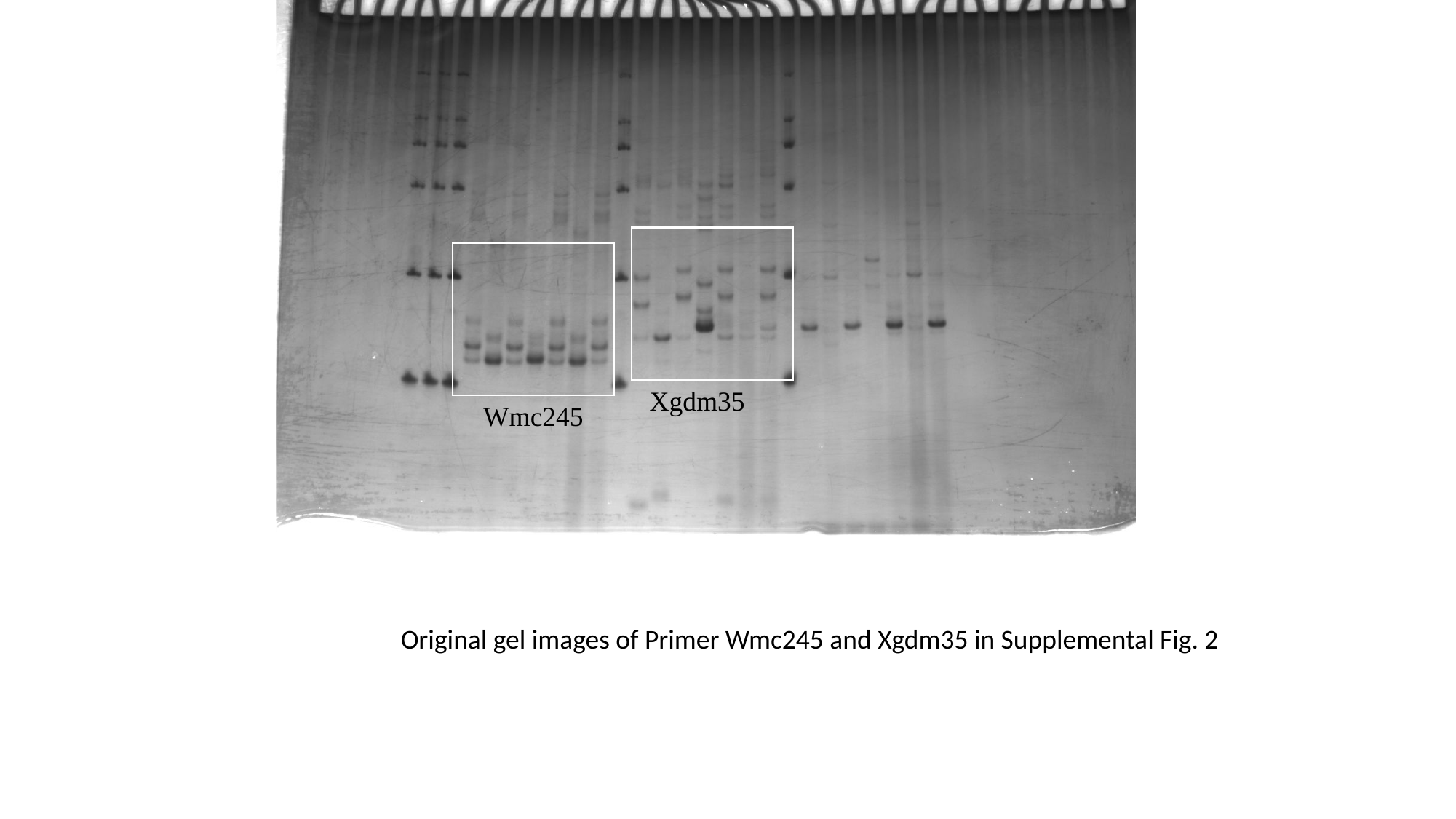

Xgdm35
Wmc245
Original gel images of Primer Wmc245 and Xgdm35 in Supplemental Fig. 2

## Slide 7
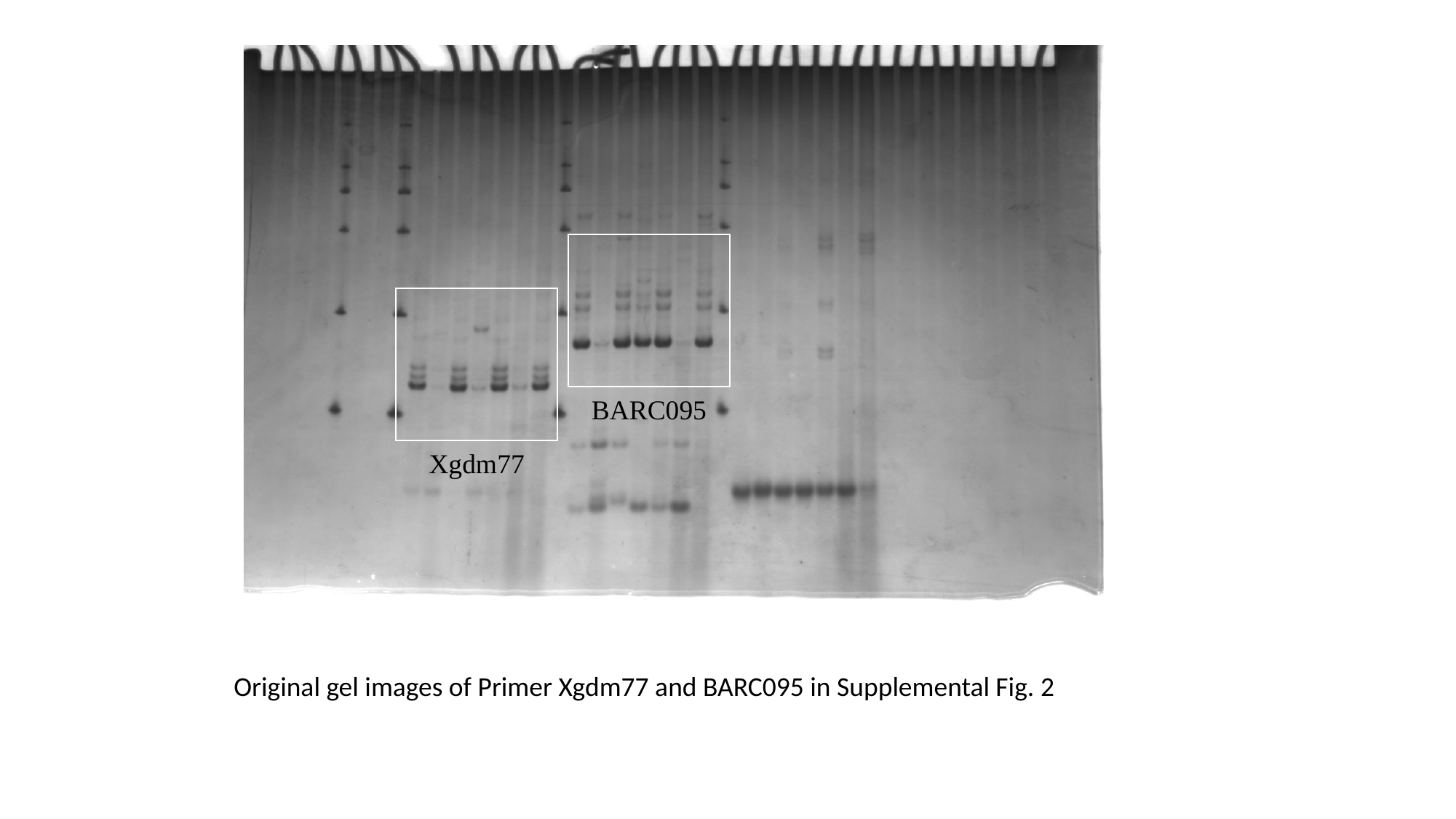

BARC095
Xgdm77
Original gel images of Primer Xgdm77 and BARC095 in Supplemental Fig. 2
