# Supplementary material for: Characterization of chromosome constitution in three wheat - Thinopyrum intermedium amphiploids revealed frequent rearrangement of alien and wheat chromosomes
Source: BMC Plant Biol. 2021 Mar 4;21:129. doi: 10.1186/s12870-021-02896-9 (PMC7931331; doi:10.1186/s12870-021-02896-9)
Supplement: Supplementary file 5 — Additional file 5: Supplemental Table 1. Chromosomal configurations of TE261–1, TE266 and TE346–1 at PMC MI. [file 12870_2021_2896_MOESM5_ESM.docx]

Supplemental Table 1. Chromosome configurations of TE261-1, TE266 and TE346-1 at PMC MI

| Material | Plants observed | Cells scored | Average chromosome configuration | | | | Relative chaotic coefficient |
| --- | --- | --- | --- | --- | --- | --- | --- |
|  |  |  | Ⅰ | Ⅱ | Ⅲ | IV |  |
| TE261-1 | 6 | 45 | 0.089 | 27.778 | 0.044 | - | 0.005 |
| TE266-1 | 5 | 40 | 0.150 | 27.85 | 0.050 | - | 0.007 |
| TE346-1 | 5 | 42 | 0.095 | 27.905 | - | 0.024 | 0.004 |

Relative chaotic coefficient (RCC) = (number of univalents + number of multivalents)/number of bivalents.
