# Supplementary material for: Characterization of chromosome constitution in three wheat - Thinopyrum intermedium amphiploids revealed frequent rearrangement of alien and wheat chromosomes
Source: BMC Plant Biol. 2021 Mar 4;21:129. doi: 10.1186/s12870-021-02896-9 (PMC7931331; doi:10.1186/s12870-021-02896-9)
Supplement: Supplementary file 6 — Additional file 6: Supplemental Table 2. Sequence alignment results for the screened wheat chromosome-specific molecular markers. [file 12870_2021_2896_MOESM6_ESM.docx]

Supplemental Table **2.** Sequence alignment results for the screened wheat chromosome-specific molecular markers

| Molecular marker | Forward/reverse | | Sequence (5'-3') | Chromosome | Initial position | Terminal position | Degree of matching |
| --- | --- | --- | --- | --- | --- | --- | --- |
| Xmag3124 | | F | ACCTAGCCAGCACATCATCC | chr1A | 593287178 | 593287161 | 94.444 |
|  |  | R | CGAGAAAGTGAGGAGGTCCA | chr1A | 593286873 | 593286855 | 94.737 |
| GPW4344 | | F | CCTGCAAGGTTTCAATTCGT | chr6A | 389958799 | 389958780 | 100 |
|  |  | R | TGAGGACCGTTGGTGTCAT | chr6A | 389958694 | 389958676 | 100 |
| GPW7465 | | F | GAGAAGCCATTAAAGCCGC | chr6A | 489511748 | 489511730 | 100 |
|  |  | R | CAGCTTGGAGACGATCAGC | chr6A | 489511527 | 489511509 | 100 |
| Xgwm219 | | F | GATGAGCGACACCTAGCCTC | chr6B | 674843297 | 674843316 | 100 |
|  |  | R | GGGGTCCGAGTCCACAAC | chr6B | 674843460 | 674843497 | 100 |
| wmc245 | | F | GCTCAGATCATCCACCAACTTC | chr2D | 186776793 | 186776772 | 100 |
|  |  | R | AGATGCTCTGGGAGAGTCCTTA | chr2D | 186776665 | 186776644 | 100 |
| Xgdm35 | | F | CCTGCTCTGCCCTAGATACG | chr2D | 13754194 | 13754175 | 100 |
|  |  | R | ATGTGAATGTGATGCATGCA | chr2D | 13753556 | 13753537 | 100 |
| Xgdm77 | | F | GACACACAATAGCCAAAGCA | chr2D | 11582137 | 11582118 | 100 |
|  |  | R | TGATGTCGGCACTATTTTGG | chr2D | 11582020 | 11582001 | 100 |
| BARC095 | | F | GGGGTGTGGTTGTTTGTAAGG | chr2D | 14032404 | 14032424 | 100 |
|  |  | R | TGCGAATTCTATATACGATCTTGAGC | chr2D | 14032568 | 14032593 | 100 |
| Ppd-D1 | | F | ACGCCTCCCACTACACTG | chr2D | 33957798 | 33957781 | 100 |
|  |  | R1 | GTTGGTTCAAACAGAGAGC | chr2D | 33957403 | 33957385 | 100 |
|  | | R2 | CACTGGTGGTAGCTGAGATT | chr2D | 33955441 | 33955422 | 100 |
| BARC053 | | F | GCGTCGTTCCTTTGCTTGTACCAGTA | chr7D | 613146874 | 613146852 | 100 |
|  |  | R | GCGCGTCCTTCCAATGCAGAGTAGA | chr7D | 613146600 | 613146579 | 100 |
| BARC172 | | F | GCGAAATGTGATGGGGTTTATCTA | chr7D | 511064337 | 511064317 | 100 |
|  |  | R | GCGATTTGATTTAACTTTAGCAGTGAG | chr7D | 511064181 | 511064158 | 100 |
